# Sex Determination in *Ceratopteris richardii* Is Accompanied by Transcriptome Changes That Drive Epigenetic Reprogramming of the Young Gametophyte

**DOI:** 10.1534/g3.118.200292

**Published:** 2018-05-02

**Authors:** Nadia M. Atallah, Olga Vitek, Federico Gaiti, Milos Tanurdzic, Jo Ann Banks

**Affiliations:** * Purdue University Center for Cancer Research; ** Department of Botany and Plant Pathology, Purdue University, West Lafayette, IN 47907; † College of Computer and Information Science, Northeastern University, Boston, MA 02115; ‡ Department of Medicine, Weill Cornell Medicine and New York Genome Center, New York, NY 10013; § School of Biological Sciences, The University of Queensland, St. Lucia, Australia

**Keywords:** sex determination, RNA-seq, Ceratopteris, gametophyte, epigenetics, gibberellin, antheridiogen, transcriptome, Genetics of Sex

## Abstract

The fern *Ceratopteris richardii* is an important model for studies of sex determination and gamete differentiation in homosporous plants. Here we use RNA-seq to *de novo* assemble a transcriptome and identify genes differentially expressed in young gametophytes as their sex is determined by the presence or absence of the male-inducing pheromone called antheridiogen. Of the 1,163 consensus differentially expressed genes identified, the vast majority (1,030) are up-regulated in gametophytes treated with antheridiogen. GO term enrichment analyses of these DEGs reveals that a large number of genes involved in epigenetic reprogramming of the gametophyte genome are up-regulated by the pheromone. Additional hormone response and development genes are also up-regulated by the pheromone. This *C. richardii* gametophyte transcriptome and gene expression dataset will prove useful for studies focusing on sex determination and differentiation in plants.

*Ceratopteris richardii* is a homosporous fern that produces a single type of haploid spore, with each spore having the potential to develop as a free-living male or hermaphroditic gametophyte. In this and many other fern species, the sex of the gametophyte is determined by a male-inducing pheromone called antheridiogen (Warne and Hickok 1991; Banks 1999). In the absence of $A_{CE}$ (for **A**ntheridiogen ***Ce***ratopteris), a spore develops as a hermaphrodite, which begins to secrete biologically detectable amounts of $A_{CE}$ after it loses the competence to respond to its male-inducing effects. In the presence of $A_{CE},$ a spore develops as a male gametophyte. Thus, in a population, spores that germinate first in the absence of $A_{CE}$ develop as hermaphrodites that secrete $A_{CE},$ while spores that germinate later and in the presence of $A_{CE}$ develop as males (Banks *et al.* 1993; Warne and Hickok 1991). Given that the self-fertilization of a hermaphroditic gametophyte results in a completely homozygous sporophyte (similar to a double haploid), this mechanism of sex determination is presumed to promote outcrossing by increasing the proportion of males in a population of gametophytes (Haufler 2002).

Although small (<3mm), male and hermaphroditic gametophytes are dimorphic and easy to distinguish by size and shape. Each hermaphrodite forms a multicellular, lateral meristem that contributes to its heart-shaped appearance, with multiple archegonia developing after the lateral meristem forms (Figure 1g). The development of this lateral meristem coincides with the loss of competence to respond to $A_{CE}$ as well as the production of $A_{CE}$ in the hermaphrodite (Banks *et al.* 1993). Male gametophytes never develop a lateral meristem and are much smaller than hermaphrodites (Figure 1d), with nearly all cells of the male gametophyte terminally differentiating as antheridia. If removed from media containing $A_{CE}$, undifferentiated cells of a male can form a new hermaphroditic prothallus. Based upon these observations, $A_{CE}$ has many functions in gametophyte development: it prevents the establishment of the lateral meristem; it promotes the rapid differentiation of antheridia; it prevents its own synthesis or secretion in the male; and is necessary to maintain the male program of expression.

**Figure 1:**
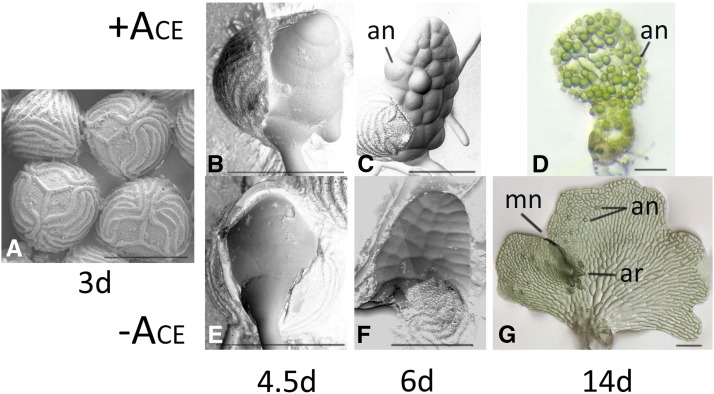
*Ceratopteris* gametophyte development. (a) SEM of spores three days after inoculation showing trilete markings. (b-d) SEMs of 4.5d, 6d and 14d gametophytes grown in the presence of $A_{CE}$. (e-g) SEMs of 4.5d, 6d and 14d gametophytes grown in the absence of $A_{CE}$. The mature hermaphrodite (g) has a meristem notch (mn), archegonia (ar) and antheridia (an) while the mature male (d) has only antheridia (an). Bars = 0.15mm.

To date, all fern antheridiogens characterized are gibberellins (GAs) (Yamane *et al.* 1987; Yamane 1998; Takeno *et al.* 1989; Furber *et al.* 1989). Although the structure of $A_{CE}$ is unknown, the GA biosynthetic inhibitors ancymidol, AMO-1618, and uniconazole-P reduce the proportion of males in a population of *Ceratopteris* gametophytes suggesting that $A_{CE}$ and GA share a common biosynthetic pathway (Warne and Hickok 1989). That ABA completely blocks the $A_{CE}$ response in *Ceratopteris* is also consistent with $A_{CE}$ being a GA (Hickok 1983; McAdam *et al.* 2016).

To understand how $A_{CE}$ determines the sex of the *Ceratopteris* gametophyte, mutations affecting sexual phenotype have been characterized and used to develop a genetic model of the sex-determining pathway (Strain *et al.* 2001; Banks 1994, 1997; Eberle and Banks 1996). Cloning these genes is challenging because of the large genome size of *C. richardii*, ca. 11Gb (Li *et al.* 2015), and the lack of a reference genome sequence for this or any other homosporous fern species. An alternative approach to identifying sex-determining genes involves *de novo* transcriptome assembly using RNA-seq, which provides a means to perform sensitive gene expression studies in organisms that do not have a reference genome (Grabherr *et al.* 2011; Robertson *et al.* 2010; Schulz *et al.* 2012). Here we describe the *de novo* assembly of the transcriptome of young *Ceratopteris* gametophytes and identify genes whose expression differs as their sex is being determined by the absence or presence of $A_{CE},$ thus providing a snapshot of the transcriptional changes that occur as the sex of the spore becomes determined and prior to the differentiation of male or female traits in the developing gametophyte.

## Materials And Methods

### Plants and Growth Conditions

The origins of Hn-n, an isogenic, wild-type strain of *Ceratopteris richardii* used in this study, is described in (Hickok *et al.* 1987). The conditions for spore sterilization and gametophyte culture are as previously described (Banks 1994). Medium used to culture gametophytes in the absence of exogenous $A_{CE}$ is as described in (Banks *et al.* 1993). and is referred to as fern medium, or FM. $A_{CE}$ was obtained as a crude aqueous filtrate from media previously supporting gametophyte growth in FM as described in (Banks *et al.* 1993) and is referred to as conditioned FM (CFM). Scanning electron micrographs (SEMs) were performed on a FEI NOVA nanoSEM on samples prepared as previously described (Banks 1994).

For both RNA-seq and qRT-PCR, spores were grown aseptically in liquid FM at 28° in a growth chamber, shaken at 100 rpm, and at a density of 1g spores/L. Three days after spore inoculation, gametophytes were filtered from media; 1/6 of the spores were added to each of three flasks containing 200 mL sterile FM, which is the $-A_{CE}$ treatment, and 1/6 were added to each of three flasks containing 200 mL sterile CFM, which is the $+A_{CE}$ treatment. After 36 hr, gametophytes were vacuum filtered from media and frozen in $N_{2}\left(l\right)$. Tissue was subsequently stored at -80°. All samples were randomized throughout incubators and during sample preparation and harvesting protocols.

### Library Preparation and Sequencing

Frozen tissue was ground under liquid nitrogen for 20 min and total RNA extracted using the RNeasy Plant Mini Kit (Qiagen, CA). The TruSeq kit (Illumina, CA) was used to select poly-adenylated mRNA and prepare libraries for sequencing. Libraries were sequenced on an Illumina HiSeq2000 platform using paired-end technology.

### Transcriptome Assembly and Annotation

DeconSeq v.0.4.1 was run on each of the FASTQ read files to remove reads aligning to bacterial, viral, rRNA, mitochondrial RNA, and chloroplast DNA (Schmieder *et al.* 2011; Schmieder and Edwards 2011). After removing adapter sequences and trimming reads based on quality score with Trimmomatic v.0.22 (Lohse *et al.* 2012), reads were assembled using the *de novo* transcriptome assembler Trinity (Grabherr *et al.* 2011), with a minimum contig length cutoff of 150 and a fixed k-mer size of 25. An assembly with unique genes was generated by selecting the longest component from each Trinity de Bruijn graph. These were used in subsequent differential expression analyses in order to avoid biasing analyses toward genes that were more difficult to assembly and thus had many more contigs (subcomponents). The program Assembly Stats in the iPlant Discovery environment was utilized to obtain basic assembly statistics (Goff *et al.* 2011; Earl *et al.* 2011). Protein-encoding, differentially expressed genes were annotated using the Trinotate workflow (Ashburner *et al.* 2000; Finn *et al.* 2011; Grabherr *et al.* 2011; Kanehisa *et al.* 2011) using a 50 amino acid minimum cutoff.

### Differential Expression Analysis

Paired reads were aligned to the assembled transcriptome using RSEM v.1.0.1 (Li and Dewey 2011; Grabherr *et al.* 2011; Li *et al.* 2015). Only the transcripts with at least one read aligned in at least one of six samples were used. edgeR v.3.0.8 (Robinson *et al.* 2010), DESeq v.1.10.1 (Anders and Huber 2010), and EBseq v.1.1.4 (Leng *et al.* 2013) were used to identify differentially expressed genes at a Benjamini-Hochberg (Benjamini *et al.* 2001) corrected FDR of q = 0.01. In edgeR, dispersion was estimated as tagwise dispersion. To retain as much rigor in our methods as possible, genes that were identified as statistically significantly differentially expressed in all three packages and displayed at least a twofold expression difference between conditions were identified as “consensus DEGs” and used in all downstream analyses.

### GO Enrichment and Assembly Validation

Because there is no reference genome sequence for *C. richardii*, GO enrichment was performed by annotating the *C. richardii* transcriptome and the differentially expressed genes against the *Arabidopsis* proteome (Araport11) (Hanlon *et al.* 2015), the non-redundant database, and the *Selaginella moellendorffii* proteome v. 1.0 (Banks *et al.* 2011), using BLASTx and an e-value threshold of $10^{-8}.$ Gene Ontology (GO) terms were then assigned from the *Arabidopsis* accession identifier from the best hit associated with the differentially expressed transcripts and the reference *C. richardii* transcriptome. ClueGO (version 2.3.4), a Cytoscape (version 3.5.1) plug-in (Cline *et al.* 2007; Smoot *et al.* 2010; Saito *et al.* 2012; Shannon *et al.* 2003), and GO Term Fusion were used to distill and visualize the GO term enrichments within the biological processes category using default parameters, with the following exceptions: the minimum number of genes/cluster was set to 5, the Benjamini-Hochberg method was used to correct the p-values for multiple testing, with a significance threshold of *P* < 0.05 and a custom background model supplied. The GO terms mapping to the entire non-redundant *Ceratopteris richardii* transcriptome was used as the background when assessing the enrichment of GO terms. To assess the quality of the *C. richardii* Trinity assembly, the 5133 *C. richardii* Sanger-generated ESTs available in GenBank were used to blast the entire *Ceratopteris* transcriptome assembly using BLASTn and a BUSCO (Benchmarking Universal Single-Copy Orthologs) analysis was performed using BUSCO v.2.0 to assess completeness of the assembled transcriptome using the ‘eukaryotic’ dataset, which consists of 303 highly conserved genes (Simão *et al.* 2015; Waterhouse *et al.* 2018).

### Expression Analysis Validation

Total RNA was isolated from six gametophyte populations cultured and harvested in the same manner as that used to generate the RNA-seq data. RNA was reverse transcribed using the Tetro cDNA Synthesis Kit (Bioline, MA); qRT-PCR was performed using the SYBR green PCR Master Mix (Applied Biosystems), 3ng cDNA template and the StepOne Real-Time PCR System (Applied Biosystems, NY). PCR conditions were: one cycle of 20 min at 95°, 40 cycles of 3 sec at 95° and 30 sec at 60°. Melt curves were analyzed and only those reactions producing a single Tm peak were used. Three technical replicates were performed for each sample. Measurements were normalized to the amount of CrEF1α (GenBank accession number BE642078) transcript in the samples. Reactions without template added served as the negative control. The Δ Ct method was used in calculating relative fold changes (Livak and Schmittgen 2001). The primer sequences used are listed in Table S1.

### Data Availability

Strains are available upon request. Table S1 contains primers used in qRT-PCR and supplemental figures. Table S3 contains a list of all 1,163 differentially expressed genes found by all three statistics packages with annotation and statistical support included. This Transcriptome Shotgun Assembly project has been deposited at DDBJ/EMBL/GenBank under the accession GBGN00000000. The version with 82,870 genes used in the differential expression analysis is the second version, GBGN02000000. RSEM results and statistical support for all Trinity predicted transcripts are available upon request. Supplemental material available at Figshare: https://doi.org/10.25387/g3.6100139.

## Results And Discussion

### Gametophyte Morphology and Selection of Tissue Samples

The early development of Ceratopteris gametophytes can be divided into distinct stages (Banks *et al.* 1993). During the first stage (0-3d after spore inoculation), the spore swells but remains intact (Figure 1a). During stage 2 (3-4d), the spore coat cracks along its trilete markings. The first rhizoid emerges from the spore during stage 3 and the two-dimensional protonema (Figure 1b and e) emerges during stage 4 (4-6d). The male and hermaphrodite gametophytes become morphologically distinct at stage 5 (6-7d; (Figure 1c and f) at which time hermaphrodites begin to secrete $A_{CE}$. For a gametophyte to develop as a male, it must continuously be exposed to $A_{CE}$ during stages 2 and 3 (Banks *et al.* 1993). Because we are interested in identifying genes that are differentially expressed by $A_{CE}$ treatment during the period of time that the sex of the gametophyte is determined, three populations of gametophytes were grown without $A_{CE}$ for three days; at day three (end of stage 1), each population was divided into two and either media without $A_{CE}$ or media with $A_{CE}$ was added to the split samples. All gametophytes were harvested and processed 36hr later (stage 3; (Figure 1b and e)) when the sex of the gametophyte was determined but male and hermaphrodites were morphologically indistinguishable.

### Transcriptome Assembly and Annotation

The *Ceratopteris* transcriptome was assembled from 188 million Illumina paired end reads generated from the six gametophyte samples (see Table S2 for a summary of run metrics, analysis and assembly of the transcriptome). Three biological replicate samples were sequenced and analyzed for each treatment condition. A Trinity (Grabherr *et al.* 2011) *de novo* assembly resulted in 82,820 genes with read support of which 24% could be annotated with the *Arabidopsis* proteome, and 23% could be annotated by the *Selaginella* proteome. A large number of genes (1,064) could be annotated using the *Selaginella* proteome but did not have hits in the *Arabidopsis* proteome. Most of the top hits of these sequences are from the *Selaginella moellendorffi* genome (302), however many are also from *Physcomitrella patens* (175), and *Marchantia polymorpha* (131). Of these, the majority (738 sequences) had hit descriptions of predicted/hypothetical, uncharacterized, or unknown proteins. That most of these sequences do not have known gene functions is not surprising given that *Arabidopsis* is generally used in annotation of plant datasets. Of the remaining sequences which were annotated, many are sperm-related. Motile sperm are a characteristic of early divergent land plants such as *Selaginella* and *Ceratotperis* (reviewed in (Hodges *et al.* 2012)), and thus it is not surprising that such proteins would be present in these assemblies but notably absent from *Arabidopsis*. A total of 44 sequences have blast hits to dynein related proteins and 18 have hits to flagellar associated proteins. Additional sequences are also present which are sperm-related, including radial spoke protein 9 and sporangia induced deflagellation-inducible protein. Of the annotated sequences, 43 are annotated with the cellular component GO term cilium, and 13 are annotated with cilium or flagellum-dependent cell motility; these are likely sperm-related proteins as flagellum are solely found in sperm cells in seedless vascular land plants (Raven *et al.* 2005).

Following the assembly and annotation of the *Ceratopteris* gametophyte transcriptome, the quality of the assembly was assessed. First, the quality of the Trinity assembly was assessed by comparing 5,133 *Ceratopteris* Sanger EST sequences available in GenBank to transcript sequences generated by Trinity using BLASTn. 87% of the Sanger ESTs, generated either from *C. richardii* sporophyte and gametophyte tissues were identical or almost identical (E-value of 0.0) to transcripts in the transcriptome assembly, indicating that Trinity accurately assembled transcript sequences from the short Illiumina reads. The expression of the Sanger ESTs not represented in the transcriptome assembly may be age or tissue specific and thus not captured in the transcriptome assembly described here. A BUSCO analysis (Waterhouse *et al.* 2018; Simão *et al.* 2015) was also performed to assess the completeness of the transcriptome. BUSCO identifies highly conserved genes as complete, complete and single-copy, fragmented, or missing in the transcriptome. Of the 303 total BUSCO groups searched, 290 were complete (95.7%), 181 were complete and single-copy (59.7%), 10 were fragmented (3.3%), and only 3 were missing (1%). This suggests that the assembled *Ceratotperis* gametophyte transcriptome is quite complete.

### Identification and Validation of Differentially Expressed Genes by Antheridiogen Treatment

**Figure 2:**
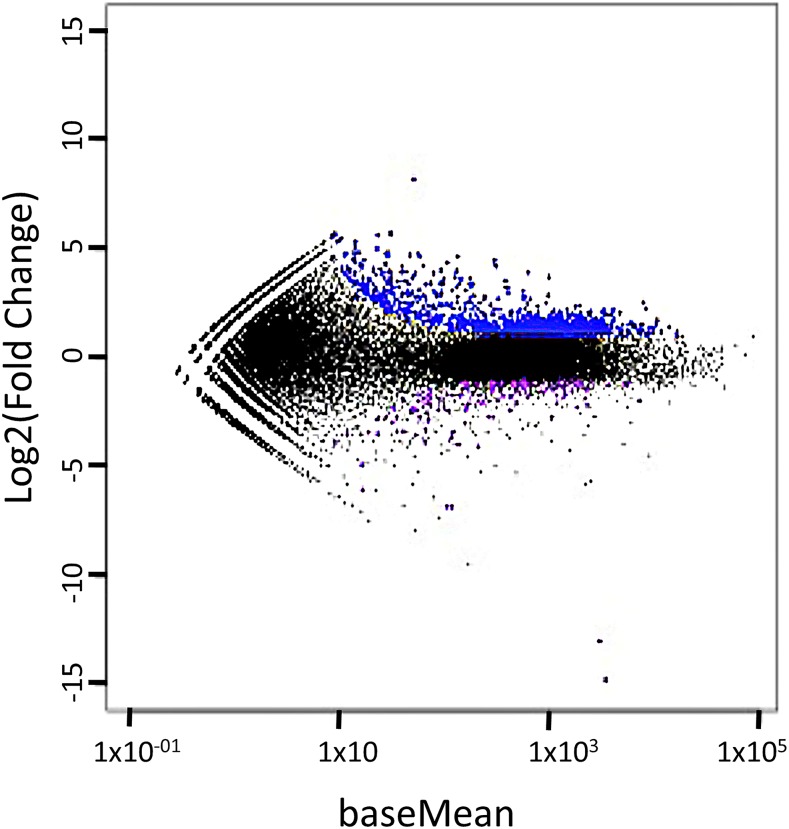
MA plot showing the $log_{2}$ Fold change *vs.* the baseMean (normalized average expression), as calculated by DESeq (Anders and Huber 2010). Genes which are more highly expressed in $+A_{CE}$ treatment are shown in blue whereas those more highly expressed in $-A_{CE}$ treatement are shown in purple. The majority (88%) of differentially expressed genes were more highly expressed in $A_{CE}$ treated gametophytes.

Three programs, edgeR (Robinson *et al.* 2010), DESeq (Anders and Huber 2010), and EBSeq (Leng *et al.* 2013), were used to identify genes that differ in their expression by $A_{CE}$ treatment (See Table S2 for number of differentially expressed genes found by each package). A scatterplot (Figure 2) that assesses the overall expression pattern across all transcripts shows that the expression of most transcripts is similar regardless of treatment, as expected. The majority (88%) of differentially expressed genes were more highly expressed in $A_{CE}$ treated gametophytes (Figure 2). Of the 1,183 DEGS identified using DESeq, 1,163 were also identified by EBSeq and edgeR; these 1,163 DEGS were used in subsequent analyses. A list of the 1,163 DEGS, their annotation and supporting statistics is provided in Table S3. Of the 133 DEGS more abundant in the non-$A_{CE}$-treated gametophytes, 55% were annotated as protein-encoding genes, while 71% of the 1,030 DEGS more abundant in the $A_{CE}$ treated samples could be annotated.

To test the validity of the DEG analysis, the expression of 10 genes, including genes more abundant in $A_{CE}$-treated samples, genes more abundant in the non-$A_{CE}$-treated samples and genes showing a less than twofold difference in abundance between treatments were assessed by qRT-PCR. As shown in Figure S1, the qRT-PCR expression data are consistent with the RNA-seq expression data in the direction of the fold change.

### GO-Enrichment of Differentially Expressed Genes

**Figure 3:**
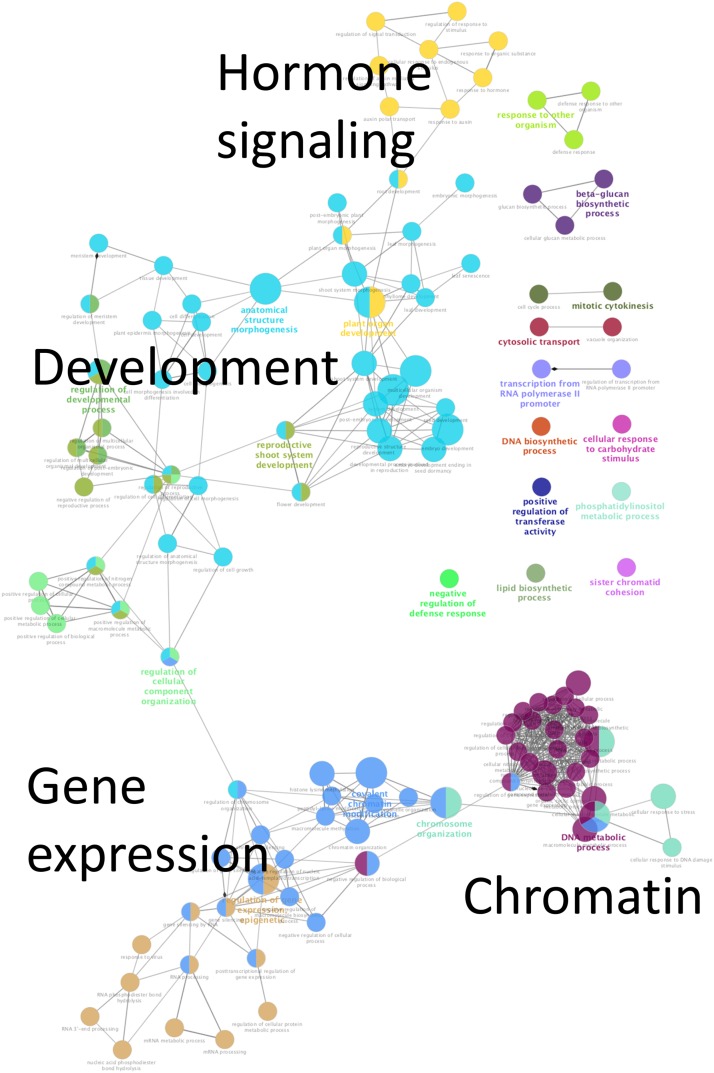
Functionally grouped Biological Process GO terms specific for $A_{CE}$-up regulated DEGs. The size of each node represents the term enrichment significance. Node labels are shown in the bar graph in Figure S2.

The enrichment of Gene Ontology (GO) Biological Process terms associated with the genes that are up-regulated by $A_{CE}$ (Figures 3 and Figure S2) reveals four major networks of enriched GO terms. One cluster includes genes related to various aspects of development, including meristem, shoot and tissue development. Another cluster includes genes involved in hormone (ABA, auxin, ethylene and GA) signaling or responses. A third cluster includes genes that affect chromatin structure and epigenetic regulation of gene expression. The fourth cluster includes genes broadly involved in regulating gene expression; genes within this cluster are included in the “chromatin” cluster. Only a single GO term (response to light stimulus) was enriched for genes that are up-regulated in the non-$A_{CE}$ treated samples.

### Hormone and Development Genes Responsive to $A_{CE}$

Given that all characterized fern antheridiogens are gibberellins (Yamane 1998), genes involved in GA hormone biosynthesis, signaling and responses are likely to be involved in sex determination in Ceratopteris. Of the differentially expressed genes, *COPALYL DIPHOSPHATE SYNTHASE/KAURENE SYNTHASE* (*CPS/KS*), which encodes a key enzyme in GA biosynthesis (Sun and Kamiya 1994; Hedden and Thomas 2012), is more abundant in gametophytes that will become $A_{CE}$ secreting hermaphrodites (Table 1). No other known GA biosynthetic genes, including *kaurene oxidase* and *GA20 oxidase* are differentially expressed in *C. richardii*, indicating that sex-specific $A_{CE}$ biosynthesis may be regulated or limited by the expression of the *CPS/KS* gene in *Ceratopteris*, and that its expression is down-regulated by $A_{CE}$ (males do not secrete $A_{CE}$). All major ABA and GA signaling genes (Yamauchi *et al.* 2004; Chan 2012; Sun 2008) are present in the *Ceratopteris* transcriptome and are listed in Table S4. *Ceratopteris* seems to have all the components seen in *Arabidopsis*, though instead of the 7 DELLA proteins, responsible for repressing GA responses, *Ceratopteris* has only 2 and two F-box protein encoding genes (*SNE* and *SLY1*) involved in GA response in *Arabidopsis* are not present in the assembled *Ceratopteris* gametophyte transcriptome. Similarities in the sex determining pathway and the GA (Atallah and Banks 2015) and ABA (McAdam *et al.* 2016; Sussmilch *et al.* 2017) signaling pathways have been described. Furthermore, three independent alleles of *Ceratopteris GAMETOPHYTE INSENSITIVE TO ABA 1* (*GAIA1*) were shown to be mutations of a gene homologous to *OST1/SLAC1* of *Arabidopsis* (McAdam *et al.* 2016). Wild type gametophytes are hermaphroditic in the presence of $A_{CE}$ and ABA, *gaia1* mutants are male in the presence of $A_{CE}$ and ABA. However, while the GA receptor (*GID*) and GA signaling (the DELLA *GAI/RGA* and *GID2*) genes are present in the transcriptome, they are not differentially expressed by antheridiogen in *C. richardii* as they are in gametophytes of the fern *Lygodium japonicum* (Tanaka *et al.* 2014). Three *MYB* genes are up-regulated (or de-repressed) by $A_{CE}$ treatment in *Ceratopteris* (Table 1). These genes are well-characterized regulators of GA-induced responses during angiosperm seed germination (Gubler *et al.* 2002) and GA-dependent anther development (Aya *et al.* 2011) and may serve similar functions by promoting the development of antheridia and/or suppressing archegonia development in *Ceratopteris* gametophytes exposed to $A_{CE}$.

**Table 1:** Differentially Expressed Genes Discussed in Text

*Ceratopteris* Gene	Annotation	*Arabidopsis* Accession	Blast E-value	AdjPval	FoldChange
**Genes more abundant in** $-A_{CE}$ **treated gametophytes**					
GA					
comp112296	copalyl diphosphate synthase	AT4G02780.1	3.00E-159	0.001459703	2.2
ABA					
comp103387	ABA 8’-hydroxylase	AT4G19230.1	0	0.003233766	2.4
comp112296	copalyl diphosphate synthase	AT4G02780.1	3.00E-159	0.001459703	2.2
comp112296	copalyl diphosphate synthase	AT4G02780.1	3.00E-159	0.001459703	2.2
comp112296	copalyl diphosphate synthase	AT4G02780.1	3.00E-159	0.001459703	2.2
CYtokinin					
comp80125	ARR9	AT2G41310.1	3.00E-42	1.18E-08	5.3
comp82535	ARR9	AT2G41310.1	2.00E-48	1.30E-08	4.2
comp119738	KAR-UP F-box 1	AT1G31350.1	4.00E-32	9.17E-05	2.3
**Genes more abundant in** $+A_{CE}$ **treated gametophytes**					
GA					
comp116986	SCARECROW-like (SCL)	AT5G66770.1	1.00E-87	0.006147273	2.4
comp82755	GRAS family transcription factor	AT1G63100.1	1.00E-92	0.000454384	2.7
comp103126	LOST MERISTEMS (LOM)	AT3G60630.1	5.00E-49	2.53E-05	2.9
comp81241	Lateral root primordium (LRP)	AT3G51060.1	2.00E-30	4.22E-06	3.6
comp42166	MOTHER of FT and TF 1 (MFT)	AT1G18100.1	5.00E-62	0.000371991	2.5
ABA					
comp82182	ARM repeat protein	AT5G19330.1	0	0.005713374	2.2
comp100365	ABA-insensitive 3 (ABI3)	AT3G24650.1	2.00E-40	0.000180668	2.6
comp103619	Protein phosphatase 2C	AT1G72770.3	1.00E-38	2.88E-05	3.2
comp114719	KEEP ON GOING (KEG)	AT5G13530.1	0	1.01E-07	3.7
Ethylene					
comp106297	ETHYLENE-INSENSITIVE2 (EIN2)	AT5G03280.1	1.00E-64	0.001387265	2.5
Auxin					
comp101920	NO VEIN (NOV)	AT4G13750.1	0	0.000253886	2.7
comp106375	PIN-FORMED 4 (PIN4)	AT2G01420.1	4.00E-166	2.68E-08	4.6
comp105872	PIN-FORMED 3 (PIN3)	AT1G70940.1	5.00E-156	0.009233133	2.2
comp98976	BIG auxin transport protein	AT3G02260.1	0	7.20E-09	4.2
comp109704	ABC transporter	AT3G28860.1	0	7.48E-12	4.7
comp97116	SART-1 family protein DOT2	AT5G16780.1	3.00E-132	0.008328934	2.5
comp114948	SAR1	AT1G33410.2	0	4.83E-05	3
comp105798	auxin response factor (ARF)	AT1G19220.1	5.00E-53	0.000175818	6.5
Cytokinin					
comp111805	AHK4; cytokinin receptor CRE1a	AT2G01830.1	0	0.000132766	3.2
comp100079	CKI1	AT2G47430.1	4.00E-108	0.000725748	2.6
DNA methylation/demethylation					
comp115365	cytosine methyltransferase (MET1)	AT5G49160.1	0	1.45E-06	3.3
comp82159	chromomethylase (CMT3)	AT1G69770.1	1.00E-155	0.006306721	2.3
comp112176	DEMETER-like protein 1 (ROS1)	AT2G36490.1	8.00E-83	0.001457903	2.7
comp101924	NERD	AT2G16485.1	7.00E-96	4.01E-05	3
Chromatin remodeling					
comp109662	CHR11; chromatin-remodeling 11	AT3G06400.2	0	0.007217854	2.2
comp83245	CHR5; chromatin remodeling 5	AT2G13370.1	0	2.28E-08	3.9
comp103550	CHR4; chromatin remodeling 4	AT5G44800.1	0.00E+00	6.59E-09	4.1
comp40502	PICKLE (PKL)	AT2G25170.1	0.00E+00	0.00059255	2.6
comp103233	PICKLE (PKL)	AT2G25170.1	5.00E-124	6.59E-05	2.8
comp39118	BRAHMA (BRM)	AT2G46020.2	0	5.18E-12	5
comp43532	CHR21/INO80	1.75E-05	3		
Histone modification					
comp81987	MBD09; methyl-CpG-binding domain	AT3G01460.1	5.00E-103	4.26E-09	4.1
comp99654	SUVH4/KYP	AT5G13960.1	0	0.001195543	2.5
comp83034	CURLYLEAF (CLF)	AT2G23380.1	0	0.000158512	2.8
comp102724	ATX2	AT1G05830.2	0	0.000231694	2.8
comp83655	ATXR3	AT4G15180.1	2.00E-180	8.34E-08	3.8
comp98691	HAC12 histone acetyltransferase	AT1G16710.1	0	0.000576165	2.6
comp62161	HAC1 histone acetyltransferase	AT1G79000.1	0	0.001018811	2.6
comp108638	HAC1 histone acetyltransferase	AT1G79000.1	0	0.00334741	2.5
comp98650	subunit of Elongator	AT5G13680.1	0	0.009770466	2.2
comp106634	ASHH2 histone-lysine N-methyltransferase	AT1G77300.2	2.00E-94	5.83E-06	3.1
comp110316	IDM1 histone H3 acetyltransferase	AT3G14980.1	1.00E-111	7.37E-05	3
comp111521	histone deacetylase HDA14	AT4G33470.1	0	0.007411904	2.3
comp109495	SUVH6	AT2G22740.1	2.00E-142	0.00720228	2.5
RNA-mediated gene silencing pathways					
comp108491	ARGONAUTE1 (AGO1)	AT1G48410.1	0	0.000891723	2.5
comp82278	ARGONAUTE1 (AGO1)	AT1G48410.1	0	0.000345422	2.6
comp112142	DICER-LIKE 1 (DCL1)	AT1G01040.1	0	0.001158223	2.5
comp110523	DICER-LIKE 1 (DCL1)	AT1G01040.1	0	0.000162621	2.9
comp37939	DICER-LIKE 4 (DCL4)	AT5G20320.1	2.00E-179	0.00411352	2.4
comp81990	THO complex subunit 2	AT1G24706.1	0	2.46E-05	3
comp82821	SOU	AT3G48050.2	3.00E-91	2.98E-08	3.9
comp81850	NRPD2a	AT3G23780.1	0.00E+00	8.85E-03	2.2
comp111720	NRPD2b	AT3G18090.1	0.00E+00	1.06E-03	2.5
comp115970	XRN4	AT5G57610.1	8.00E-148	2.28E-07	2.9

Although ferns never evolved seeds, there are interesting physiological parallels between seed germination in *Arabidopsis* and sex determination in *Ceratopteris* in that both processes are regulated by two antagonistic hormones, ABA and GA. In germinating *Arabidopsis* seeds, the expression of *Mother of FT and TFL* (*MFT*) gene is modulated by GA and ABA (Xi *et al.* 2010). *MFT* is up-regulated by ABA treatment via the ABA-INSENSITIVE3 (ABI3) and ABI5 transcription factors, as well as by DELLA proteins in the GA signaling pathway. Because MFT represses *ABI5*, MFT is central to a negative feedback loop that regulates seed germination by GA and ABA in *Arabidopsis*. Given that the *Ceratopteris MFT* and *ABI3* genes are upregulated by $A_{CE}$ and ABA antagonizes the $A_{CE}$ response (Hickok 1983; McAdam *et al.* 2016; Sussmilch *et al.* 2017), $A_{CE}$ may promote male development by ultimately repressing ABA signaling in the gametophyte through a pathway that involves *MFT* and *ABI3*.

Several additional genes involved in ABA, ethylene, auxin and cytokinin perception, signaling or response are up-regulated by $A_{CE}$ treatment (Table 1). While ABA is known to affect sex determination by blocking the $A_{CE}$ response, these results point to roles for additional hormones in the sex-determining process. Studies of the effects of exogenous auxin (Gregorich and Fisher 2006; Hickok and Kiriluk 1984), ethylene (Kaźmierczak 2010) and cytokinin (Menéndez *et al.* 2009) on fern gametophyte development have shown that these hormones can affect the overall size and organization of the gametophyte as well as the number of sex organs in a gametophyte. However, neither auxin, ethylene or cytokinin substitute for or completely block the male-inducing effects of antheridiogen, indicating that $A_{CE}$ may influence these hormones, or the crosstalk among these hormones, in modulating cell division and expansion in young gametophytes that will become important as they differentiate.

This DEG analysis suggests that $A_{CE}$ affects the sex of the gametophyte by not only activating genes associated with development, but also by epigenetically reprogramming the nucleus that will divide and ultimately give rise to a male gametophyte. The relatively few genes that are up-regulated in gametophytes not treated with $A_{CE}$ likely represent genes that are normally expressed in the gametophyte destined to become hermaphrodite but are repressed by $A_{CE}.$

### An Epigenetic Response to $A_{CE}$

A striking number of DEGs up-regulated by $A_{CE}$ encode factors involved in epigenetic regulation of gene expression or epigenetic reprogramming of the genome. These genes were sorted into five groups (Table 1) following the classification of Pikaard and Scheid (Pikaard and Scheid 2014): DNA modification, histone modification, Polycomb-group proteins and interacting components, chromatin formation/chromatin remodeling and RNA silencing.

The first group includes DNA modification genes that affect cytosine methylation. The DEGs assigned to this group encode *DNA METHYLTRANSFERASE 1* (*MET1*), which maintains CpG methylation (Saze *et al.* 2003; Jullien *et al.* 2012), *CHROMOMETHYLASE 3* (*CMT3*), which maintains CpHpG methylation (Law and Jacobsen 2010) and *REPRESSOR OF SILENCING 1* (*ROS1*), a cytosine demethylase (Gong *et al.* 2002). Differences in global DNA methylation patterns between gametes and adjacent cells of both male and female gametophytes of *Arabidopsis* have been observed (Pillot *et al.* 2010; Calarco *et al.* 2012; Ibarra *et al.* 2012; Jullien *et al.* 2012) and are thought to silence transposable elements and reset silenced imprinted genes in sperm cells (Kawashima and Berger 2014; Martínez *et al.* 2016). While sex determination in a homosporous fern, which occurs during the gametophyte generation, differs from sex determination in the heterosporous angiosperms, which occurs during the sporophyte generation (Tanurdzic and Banks 2004), the up-regulation of these genes during sex determination in *Ceratopteris* adds another stage of plant development where DNA methylation may play an important role in stabilizing or destabilizing transposable elements and contributes to epigenetic reprogramming of the male gametophyte. Whether the observed differential expression of these DNA methylation genes alters DNA methylation patterns in the genomes of young *Ceratopteris* gametophytes, and whether additional changes in DNA methylation occur as their gametes differentiate, remain to be tested.

A number of $A_{CE}$-up-regulated DEGs encode proteins belonging to the second group, histone-modifying enzymes known to affect gene expression (Table 1). Among them are the histone acetyltransferases HAC1, HAC12 and ROS4, a histone deacetylase (HDA14), the histone methyltransferases TRITHORAX-LIKE PROTEIN 2 and 3 (ATX2 and 3), the SU(VAR)3-9 related proteins SUVH4/KYP and SUVH6, and EARLY FLOWERING IN SHORT DAYS (EFS/SDG8). These proteins are involved in either maintaining transcriptionally active states or transcriptionally inactive states (reviewed in (Liu *et al.* 2010; Bannister and Kouzarides 2011; Grossniklaus and Paro 2014; Pikaard and Scheid 2014; Xiao *et al.* 2016) and can contribute to the maintenance of DNA methylation at silenced loci. ATXR3 is notable in that it is essential for male and female gametophyte development (Berr *et al.* 2010) in angiosperms. Only one DEG, *CURLYLEAF* (*CLF*), was classified as encoding proteins from the third group of chromatin modifiers: Polycomb proteins. Polycomb proteins and interacting partners are often involved in determining cell proliferation and identify through methylation and chromatin compaction (Grossniklaus and Paro 2014; Kingston and Tamkun 2014). The fourth group of genes, those involved in chromatic formation/remodeling, are also represented among the genes up-regulated in response to $A_{CE}$. *PICKLE* (*PKL*), the gene encoding for a chromatin remodeling factor which is necessary for gibberellin modulated development in *Arabidopsis*, (Park *et al.* 2017) and INOSITOL-REQUIRING 80 (INO80), are both members of remodeling complexes and are required for normal development (Zhang *et al.* 2015).

The fifth group of genes involving epigenetic regulation is those relating to RNA-mediated gene silencing pathways (Table 1). Argonaute (AGO) 1, a core member of the RNA-induced silencing complex (RISC) which is involved post transcriptional gene silencing (PTGS) through cleavage or transcriptional inhibition (reviewed in (Czech and Hannon 2011; Martienssen and Moazed 2015)) is significantly up-regulated by $A_{CE}$. Also up regulated are genes encoding two Dicer endonucleases: DCL1 which generates miRNAs of mostly 21nt and DCL4, which generates siRNAs that are 21nt (Pouch-Pélissier *et al.* 2008). Additional genes involved in RNA-mediated PTGS are *XRN4*, which encodes a nuclease involved in small RNA processing (Cao *et al.* 2014), and *SUO*, which encodes a component of the miRNA pathway (Yang *et al.* 2012). *NRPD2*, encoding the catalytic subunit of RNA polymerase IV and V in plants (Ream *et al.* 2009) is also up-regulated by $A_{CE}$. Pol IV and V are both required for intercellular RNA interference and are involved in PTGS maintenance (Onodera *et al.* 2005; Pontier *et al.* 2005; Kanno *et al.* 2005). Also modulated by $A_{CE}$ is a component of the THO/TREX complex, which has a putative role in siRNA biosynthesis (Furumizu *et al.* 2010). Interestingly, the THO complex represses female germline specification in *Arabidopsis* (Su *et al.* 2017). Together, these results show that small RNA-mediated PTGS is involved in the suppression of female characteristics in *C. richardii* gametophytes.

All of the epigenetic mechanisms known to occur in plants are represented among the genes up regulated by $A_{CE}$. The importance of epigenetic regulation for sex determination in *C. richardii* should perhaps not come as a surprise. Gametophytes which are removed from $A_{CE}$ containing media will over time develop into hermaphrodite gametophytes, thus the promotion of male/suppression of female traits must be reversible. Epigenetic regulation of sex determination would allow for such plasticity in development.

### Conclusions

This work reports the first transcriptome of *Ceratopteris richardii*, along with a survey of significant differential gene expression changes between male and hermaphrodite gametophytes as sex is being determined. A high-quality reference gametophyte transcriptome was assembled and used in the identification of genes which may be involved in sex determination. The majority of differentially expressed genes were more highly expressed in the male gametophyte. Many of these up regulated genes are known to be involved in development and in response to hormones. A significant number of differentially expressed genes are involved in chromatin remodeling and epigenetic regulation. Outcomes of this research shed light on the molecular mechanisms involved in sex determination of *C. richardii* as well as provide a resource for other plant science researchers. Future work will probe and functionally classify these differentially expressed genes and as well as survey how these changes persist as the gametophyte moves from sex determination to differentiation.
